# Consistent trends from different methods for monitoring SARS-CoV-2 in urban wastewater during a 29-month longitudinal study

**DOI:** 10.3389/fmicb.2025.1547831

**Published:** 2025-06-20

**Authors:** Janine McCalder, Jangwoo Lee, Judy Qiu, Qiaozhi Li, Linnet Immaraj, Nicole Acosta, María A. Bautista, Melissa Wilson, Barbara Waddell, Kristine Du, Aito Ueno, Rhonda Clark, Alexander Krusina, Danielle A. Southern, Tyler Williamson, Chloe Papparis, Paul Montesclaros, Lance Non, September Stefani, Gail Visser, Puja Pradhan, Norma Ruecker, John Conly, Steve Hrudey, Kevin Frankowski, Bonita Lee, Michael D. Parkins, Xiaoli Pang, Casey R. J. Hubert

**Affiliations:** ^1^Geomicrobiology Group, Department of Biological Sciences, University of Calgary, Calgary, AB, Canada; ^2^Department of Microbiology, Immunology and Infectious Diseases, University of Calgary, Calgary, AB, Canada; ^3^Department of Laboratory Medicine and Pathology, University of Alberta, Edmonton, AB, Canada; ^4^School of Public Health, University of Alberta, Edmonton, AB, Canada; ^5^Advancing Canadian Water Assets, University of Calgary, Calgary, AB, Canada; ^6^Department of Medicine, Cumming School of Medicine, University of Calgary, Calgary, AB, Canada; ^7^Department of Community Health Sciences, University of Calgary, Calgary, AB, Canada; ^8^O’Brien Institute for Public Health, University of Calgary, Calgary, AB, Canada; ^9^Department of Biological Sciences, University of Calgary, Calgary, AB, Canada; ^10^Water Quality Services, Calgary, AB, Canada; ^11^Department of Pathology and Laboratory Medicine, University of Calgary, Calgary, AB, Canada; ^12^Infection Prevention and Control, Alberta Health Services, Calgary, AB, Canada; ^13^Snyder Institute for Chronic Diseases, University of Calgary, Calgary, AB, Canada; ^14^Analytical and Environmental Toxicology, University of Alberta, Edmonton, AB, Canada; ^15^Department of Pediatrics, University of Alberta, Edmonton, AB, Canada; ^16^Women and Children’s Health Research Institute; Li Ka Shing Institute of Virology, Edmonton, AB, Canada; ^17^Alberta Precision Laboratories, Public Health Laboratory; Li Ka Shing Institute of Virology, Alberta Health Services, Edmonton, AB, Canada

**Keywords:** SARS-CoV-2, wastewater, wastewater-based epidemiology, concentration method, direct extraction, method comparison, affinity column, ultrafiltration

## Abstract

Rigorous method development and validation to detect and quantify SARS-CoV-2 RNA in wastewater has led to important advances in community disease surveillance using quantitative molecular biology tools. Despite this progress, agreement on standardized workflows for this important public health objective has been elusive. Multiple studies have compared different protocols but have been limited by short periods of observation or low numbers of test sites. Here we compare results from two parallel workflows for wastewater processing and quantifying SARS-CoV-2 gene targets from five wastewater treatment plants in three large cities in Alberta, Canada for up to 29-months. In total 1,482 wastewater samples were processed using either affinity columns followed by RT-qPCR with DNA-based standards or using ultrafiltration followed by RT-qPCR with RNA-based standards. Results from either workflow correlated well with each other, and with 5-day rolling averages of clinically diagnosed COVID-19 cases (i.e., in the early part of the 29-month study period when clinical testing was performed routinely). This highlights that different workflows both effectively and reliably monitored SARS-CoV-2 trends in wastewater. Parallel quantification of pepper mild mottle virus genomes and normalization were inconsistent between the two workflows, suggesting that normalization strategies may require adjustment for different wastewater processing protocols. Freezing wastewater samples diminished measured SARS-CoV-2 RNA levels significantly, whereas short term sample storage at +4°C gave consistent results. Overall, this work demonstrates that different workflows can deliver similarly effective wastewater-based surveillance for community COVID-19 burden. As this emerging technology is used more routinely, investigators should prioritize consistent application of a given workflow to a high-quality standard over time, whereas focusing on all testing programs adopting identical workflows and methods may be unnecessary.

## 1 Introduction

Severe acute respiratory syndrome coronavirus 2 (SARS-CoV-2) is excreted in the stool of 14–50% of infected individuals (Parasa et al., 2022) allowing its quantification in community wastewater to provide an estimation of disease burden in populations contributing to corresponding sewershed catchments (Maslennikov et al., 2021; Shang et al., 2020). Wastewater-based surveillance (WBS) has been shown to effectively complement clinical testing by providing unbiased monitoring of both asymptomatic and symptomatic COVID-19 infections in an extremely cost-effective way (Ahmed et al., 2021; Farkas et al., 2020; Kitajima et al., 2020; Medema et al., 2020; Pang et al., 2022; Rimoldi et al., 2020; Torii et al., 2021). The information provides a leading measure of COVID-19 burden, as opposed to the lagging indicator of clinical test data that is not always available (Kaplan et al., 2021). Early in the COVID-19 pandemic, the lead-time associated with wastewater testing was reported as being around 4–6 days (Galani et al., 2021; Peccia et al., 2020). Sampling at wastewater treatment plants (WWTPs) allows for accurate estimation of COVID-19 burden at the broadest community level due to the large catchment areas and populations served. Sampling can take place further upstream from a WWTP to determine more granular data for smaller sub-catchments of a given sewershed, including distinct neighborhoods (Acosta et al., 2022) or individual buildings such as hospitals (Acosta et al., 2021) and long-term care facilities (Lee et al., 2021), but correlations to population-level health data have been observed to be strongest for samples from community-wide WWTPs (Acosta et al., 2022).

Quantitative detection of SARS-CoV-2 genomic RNA from wastewater samples generally requires an enrichment step due to the large dilution of viruses in wastewater relative to other nucleic acid genomic material (e.g., derived from humans and microorganisms). Various methods have been developed to selectively concentrate viral particles including polyethylene glycol precipitation, charged membrane filtration and ultrafiltration, among others (Chang et al., 1981; Falman et al., 2019; Lewis and Metcalf, 1988; Winona et al., 2001). Most of these techniques were originally developed to capture non-enveloped enteric viruses, whereas many human respiratory viruses including SARS-CoV-2 have an exterior lipid envelope (Nichols et al., 2008). At the onset of the COVID-19 pandemic, it was unknown how concentration techniques typically used for non-enveloped viruses would perform for enveloped viruses due to different capsid structures. Rigorous comparison of methods was performed for many different concentration techniques specifically to assess SARS-CoV-2 recovery. These approaches contrast with direct extraction of RNA from wastewater achieved by other methods such as “4S” using affinity column purification that was developed early in the COVID-19 pandemic (Whitney et al., 2021).

While many studies have reported the comparative performance of a wide range of wastewater sample processing methods for SARS-CoV-2 since the start of the COVID-19 pandemic (Ahmed et al., 2020; Ahmed et al., 2021; Pérez-Cataluña et al., 2021; Philo et al., 2021), these studies tend to report limited numbers of samples tested over relatively short time periods (LaTurner et al., 2020; Philo et al., 2021; Zheng et al., 2022). On the other hand, studies that have sustained regular monitoring for longer periods of time (>1 year) do not tend to prioritize side-by-side comparison of different workflows (Li et al., 2022; Pang et al., 2022). Comprehensive longitudinal comparisons of different nucleic acid isolation and quantification workflows tailored for SARS-CoV-2 WBS are needed to better inform ongoing discussions regarding whether standardized methodology should be adopted in this rapidly developing field of science. Prolonged comparison over time also allows the study of the effects of a wide range of extrinsic factors longitudinally with the purpose of appraising assay performance (e.g., ambient temperature in settings with significant seasonal variation).

To address these issues, this study presents an extensive 29-month dataset that examines and compares two fundamentally different workflows for SARS-CoV-2 surveillance. The two workflows were launched independently from each other early on in the pandemic without coordination between the two teams. Three large cities in Alberta, Canada were monitored three times per week using both affinity column- and ultrafiltration-based workflows, allowing results to be compared with each other and with available clinical testing results during the first several waves of the COVID-19 pandemic in Alberta up until November 2022.

## 2 Materials and methods

### 2.1 Establishing SARS-CoV-2 WBS throughout Alberta

Shortly after the onset of the COVID-19 pandemic, wastewater testing was initiated on samples from the three WWTPs serving Calgary, Alberta, Canada and its surrounding communities serving a total population of 1.5 million people (Urban Systems, 2019). WWTP sample splits were provided to the University of Alberta and University of Calgary, with monitoring starting in May 2020 and June 2020, respectively. Wastewater entering these WWTPs combines urban and industrial sources, whereas stormwater runoff (e.g., snow melt; rainwater) is handled via a separate drainage system and not included. All three WWTPs were monitored using both workflows up until September 2021, after which only the largest (WWTP 1) continued to be sampled for comparison purposes until November 2022 (29 months). Wastewater splits for comparison purposes were also initiated for Alberta municipalities Lethbridge and Fort McMurray in October 2021. Lethbridge and Fort McMurray WWTPs represent smaller catchments, with corresponding populations of 101,799 (Alberta, 2021) and 76,006 (Municipal Census Report, 2021), respectively. Lethbridge and Fort McMurray are the largest communities in southern and northern Alberta, respectively, and also separate wastewater and stormwater systems. SARS-CoV-2 WBS for these additional two WWTPs was initiated in 2020 at the University of Alberta, with analysis at the University of Calgary commencing in October 2021, hence for the purpose of this comparative study Lethbridge and Fort McMurray samples were analyzed for the period October 2021 to November 2022. The geographic distribution of the 5 WWTPs is shown in the map in Supplementary Figure S1.

Calgary samples collected and compared between June 2020 and January 2021 included an additional comparison of pre-frozen wastewater processed via ultrafiltration with unfrozen wastewater processed via affinity columns. After January 2021 none of the samples were pre-frozen.

### 2.2 Sample collection and handling

Raw influent wastewater samples from the three Calgary locations (WWTPs 1–3) were collected using ISCO autosamplers (Teledyne ISCO, United States) programmed to collect 80 mL of wastewater every 15 min over the course of 24 h to generate 24-h composite samples corresponding to a given sampling date. City of Calgary personnel deployed ISCO autosamplers and collected and divided each sample for shipment to the two processing laboratories at the University of Calgary and the University of Alberta (in Edmonton; approximately 3 h driving distance from Calgary). For shipping, subsamples were collected as 2 L volumes from the composite wastewater and immediately placed in coolers with ice packs. Samples were obtained and shipped up to three times per week. From June 2020 to January 2021, samples were frozen for shipment to the University of Alberta laboratory; this protocol was changed to cold (+4°C) shipping as of January 2021. Raw influent wastewater was also collected as 2 L subsamples from 24-h composite samples at the WWTPs servicing Lethbridge (WWTP 4) and Fort McMurray (WWTP 5) programmed in a similar way. Replicate samples from Lethbridge and Fort McMurray were kept cold (never frozen) and shipped to the two laboratories in Calgary and Edmonton within 72 h of collection.

### 2.3 Wastewater sample processing methods

**FIGURE 1:**
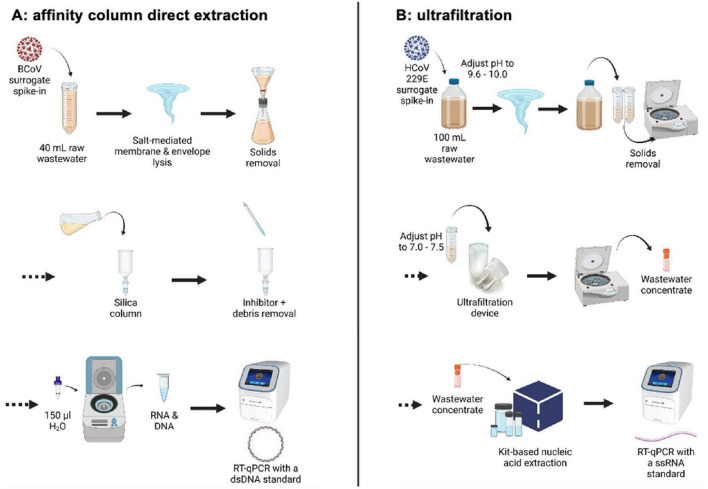
Comparative schematic of **(A)** affinity column and **(B)** ultrafiltration wastewater processing workflows. Created with BioRender.com.

Early in the pandemic, different workflows in the two laboratories were established for wastewater processing and SARS-CoV-2 RNA isolation and quantification (Figure 1). Several months later, after routine operations had been established, the two teams began to compare results, enabling this study. Workflow A at the University of Calgary used an affinity column method based on the “4S” direct extraction protocol (Whitney et al., 2021). Workflow B at the University of Alberta used an ultrafiltration method of concentration that is widely employed for recovery of viruses, including SARS-CoV-2, from different aqueous samples including wastewater, recreational water and agricultural run-off (Forés et al., 2021; Lee et al., 2021; Qiu et al., 2016; Qiu et al., 2022; Winona et al., 2001). RNA isolated using either approach was then analyzed for SARS-CoV-2 genomic quantification using the same RT-qPCR assays targeting two loci in the nucleocapsid gene (N1 and N2) (Acosta et al., 2021; Qiu et al., 2022), but with DNA standards for RT-qPCR applied in Workflow A and RNA standards applied in Workflow B. Both laboratories additionally incorporated an assay for pepper mild mottle virus (PMMoV), a plant virus found abundantly in human feces (Zhang et al., 2005), as a prospective fecal biomarker to test normalization of results against a signal representing the entire population contributing to the sewershed.

#### 2.3.1 Workflow A: affinity column isolation of RNA from wastewater at the University of Calgary

Upon arrival, samples were stored at 4°C and processed within 24 h. All samples were spiked with a bovine coronavirus (BCoV) surrogate (Merck catalogue #151921; Merck Animal Health, United States) attenuated live vaccine as an exogenous process control (2500 TCID_50_/mL) as described elsewhere (Acosta et al., 2021). From 2 L wastewater samples, a 40 mL subsample was collected using a 50 mL pipette after vigorous shaking to suspend wastewater solids. Subsamples were then aliquoted into 50 mL plastic tubes prefilled with 9.5 g sterile NaCl and 400 μL TE buffer, and then spiked with 200 μL (Ci 5 × 10^5^ TCID_50_/mL) of the BCoV surrogate. Samples were then vortexed vigorously for 30 s and large solid particles were filtered out using a vacuum filtration apparatus equipped with a 5 μm PVDF filter. Filtrate was collected directly into an equal volume of 70% ethanol and combined into solution which was then passed through a Zymo-Spin III-P silica column attached to a custom-built vacuum manifold. Two wash buffers were used consecutively to remove inhibitors and cell debris from the samples. Solutions were removed from the silica column and centrifuged at 10,000 × g for 2 min to remove any residual wash buffer. Purified nucleic acids were then eluted into 100 μL of molecular grade water preheated to 50°C and pipetted directly onto the packed silica in the columns. After 2 min incubation at 50°C, columns were centrifuged at 10,000 × g for 5 min. Eluted samples were frozen immediately at −80°C. This procedure co-elutes DNA and RNA, such that the eluent was used directly in RT-qPCR assays without additional steps (Figure 1A; Acosta et al., 2021, 2022).

#### 2.3.2 Workflow B: wastewater concentration by ultrafiltration and subsequent RNA extraction at the University of Alberta

All samples processed by ultrafiltration were spiked with an exogenous cultured human coronavirus (Qiu et al., 2016, 2022). Human coronavirus (HCoV) 229E was purchased from ATCC (VR-740) and propagated in MRC-5 cells. Aliquots of cultured virus stock were stored at −80°C. Each wastewater sample was spiked with 100 μL of diluted HCoV 229E virus (1:1,000 dilution; final concentration of 4.25 × 10^5^ TCID_50_/mL). For frozen samples collected between June 2020 and January 2021, aliquots were allowed to thaw at room temperature overnight for processing the next day. After January 2021 samples were no longer frozen during or after shipping and were kept at 4°C upon receipt and processed within 72 h. A 100 mL aliquot of the sample was mixed and transferred to a clean bottle and then spiked with 100 μL of the HCoV 229E viral surrogate. The pH was adjusted to between 9.6 and 10.0 using 5 N NaOH, and samples were vigorously shaken by hand or vortexed for 30 s. The 100 mL sample was then aliquoted into two 50 mL plastic tubes and centrifuged at 4,500 × g for 10 min for debris removal. Supernatant pH was then adjusted to between 7.0 and 7.5 with 1.2 N HCl prior to transferring 70 mL to a Centricon^®^Plus-70 centrifugal filter unit (Merck Millipore, Germany) with a molecular weight cut-off of 30 kDa. Filter units were centrifuged at 3,000 × g for 10 min at 4°C. After discarding the filtrate, the remaining 30 mL of the sample was added to the Centricon unit and centrifuged once more under the same conditions. Upon discarding the remaining filtrate, the collection cup was attached to the filter cup, carefully inverted, and centrifuged at 800 × g for 2 min. PBS was added to the concentrate to adjust all samples to a final volume of 1 mL, which was stored at −70°C until RNA extraction.

Total nucleic acid extraction following ultrafiltration was performed using the MagMAX™ Viral RNA Isolation kit with the KingFisher™ Flex Purification system (ThermoFisher Scientific, United States). A 200 μL aliquot of the viral concentrate was added to a 96-deep-well processing plate containing 510 μL of lysis buffer, 20 μL of bead mix, 2 μL of carrier RNA, and 5 μL of salmon DNA. After concentrate addition, the plate was placed in the KingFisher™ Flex for RNA extraction following manufacturer’s instructions. Beads were allowed to dry for 1 min, and the RNA was eluted into 50 μL of elution buffer over the course of 4 min. RNA purified in this way can be used in RT-qPCR assays (Qiu et al., 2022).

### 2.4 RT-qPCR detection of SARS-CoV-2 and surrogate targets

The affinity column (University of Calgary) and ultrafiltration (University of Alberta) laboratories employed RT-qPCR assays with the same primer and probe target sequences for N1, N2 and for PMMoV (Centers for Disease Control Prevention [CDC], 2020; Haramoto et al., 2013; Zhang et al., 2005; Supplementary Table S1). In addition to common N1, N2, and PMMoV targets, each workflow also implemented assays to quantify their respective exogenous viral spike-in control (Decaro et al., 2008; Vijgen et al., 2005). Certain aspects of the RT-qPCR protocols differed in Workflows A and B. Most notably, RNA that was purified using affinity columns was subsequently quantified using a double stranded plasmid DNA standard during RT-qPCR, whereas RNA extracted following ultrafiltration was quantified using a single stranded RNA standard. There were also slight differences in cycling conditions and assay volumes. For example, in Workflow A for RNA purified using affinity columns the RT-qPCR reaction volume was 20 μL, whereas in Workflow B for RNA extracted after ultrafiltration the reaction volume was 10 μL. Details about the protocols, primers, probes, and standard curve reagents can be found in Supplementary Tables S1–S13.

For Workflow A samples processed using affinity columns, N1 and N2 targets were assessed using separate one-step TaqMan™ assays. One-step multiplex TaqPath™ assay was used for simultaneous detection and quantification of PMMoV and BCoV targets. All assays were run in triplicate. Three replicates of UltraPure™ DNase/RNase free distilled water were used as a non-template control within each plate of PCR reactions, and each point of the DNA standard curve was also run in triplicate. All plates were run on a QuantStudio 5 (Applied Biosystems, United Kingdom), up until September 2022 after which a QuantStudio 6 (Applied Biosystems, United Kingdom) was used. The cycle at which the fluorescence intersects with the detection threshold is the quantification cycle (Cq). Standard curves were used to generate fluorescence data which were then used as a reference for quantification of the unknown samples. Samples were considered positive for SARS-CoV-2 if either the N1 or N2 target amplification passed the detection threshold in <40 cycles. The Cq value of a target was used to determine gene abundance if it was < 40, or between 40 and 45 if the Cq of the other target was < 40. The median RT-qPCR efficiency was 104.5% for N1 and 99.5% for N2 (standard curve slopes −3.2 to −3.3 and R^2^ 0.97–0.98). The limit of detection and limit of quantification were determined as the lowest values with a relative repeatability standard deviation among replicates of ≤ 33% and ≤ 25%, respectively, and were both 5 genome copies μL^–1^.

For Workflow B samples processed using ultrafiltration followed by RNA extraction, the N1, N2, PMMoV, and HCoV229E targets were each assessed using separate one-step TaqMan™ assays. These were run in duplicate using an ABI 7500 PCR instrument (Applied Biosystems, United Kingdom). Samples were considered positive for SARS-CoV-2 if at least 50% of the technical replicates between the N1 and N2 targets achieved Cq values < 40. To control for inter-run variability, prior samples with known Cq values were included in each run to confirm variation was < 1 Cq relative to expectations. Assay sensitivity was assessed as the lowest value by 10-fold serial dilutions (1.66 × 10^0^ to 1.66 × 10^6^ copies) of the viral RNA fragment in 10 replicates using probit logistic regression analysis. The efficiency of this RT-qPCR assay was greater than 98%. The limit of detection was 1.6 genes per PCR reaction, corresponding to 80 genome copies in 100 ml of wastewater.

### 2.5 Clinically diagnosed cases of COVID-19 during the study period

All clinical testing for COVID-19 in Alberta, Canada was performed by a single integrated health service provider, Alberta Precision Laboratories, with data reported to Alberta Health Services and Alberta Health. Data relating to the total number of COVID-19 cases reported per day corresponding to the sewershed catchment of each WWTP were extracted from the Data Analytics group of Alberta Health Services, as described elsewhere (Acosta et al., 2022). This used linked postal code data for Calgary and Local Geographic Areas for Lethbridge and Fort McMurray. Six different COVID-19 waves in Alberta during the study period were defined by Alberta Health Services and Alberta Health public health surveillance teams based on active COVID-19 case numbers as follows: 1st wave (March 8 to July 10, 2020), 2nd wave (Alpha; July 10, 2020 to February 25, 2021), 3rd wave (February 25 to July 1, 2021), 4th wave (Delta; July 1 to December 12, 2021), 5th wave (Omicron BA.1; December 12, 2021 to March 6, 2022), 6th wave (Omicron BA.2; March 6 to June 29, 2022) (Detsky and Bogoch, 2022; Geary et al., 2023). Molecular assays were able to detect all of the variants present during these waves.

### 2.6 Defining municipal populations corresponding to each WWTP catchment

WWTP-1 is the largest wastewater treatment plant in Calgary and processes the majority of wastewater generated in northern and central parts of the city, as well as several satellite communities (Airdrie, Cochrane, Cochrane Lake, Elbow Valley and Tsuu T’ina North). This catchment corresponds to a population of 1.1 million. WWTPs 2 and 3 both collect wastewater from southern Calgary and the satellite communities of Chestemere and Tsuu T’ina South corresponding to a total population of ∼363,000 (Acosta et al., 2022; Urban Systems, 2019). Wastewater can be pumped between WWTP 2 and WWTP 3 such that they are considered one system servicing the south catchment of Calgary (Urban Systems, 2019). Samples from both WWTPs 2 and 3 were included in this study to enable a more comprehensive comparison of both workflows. Wastewater from all residential areas and businesses in Lethbridge (population 101,799) is processed by a single WWTP (WWTP 4) (Wastewater Treatment Process, 2023). Fort McMurray’s WWTP (WWTP 5) services a population of 76,006 and has a catchment that is divided into two sections; the north catchment composed of 14 areas and covering an area of 7,600 ha, and the south catchment composed of 12 areas and covering an area of 5,600 ha (Wastewater Master Plan, 2014).

### 2.7 Ambient air temperature

During 24-h composite sample collection from WWTPs 1–3 in Calgary, wastewater collected by the autosamplers was removed from the passing flow in the sewer and was thus exposed to ambient temperature during its storage in-place for up to 24 h. The temperature in the underground utility holes was not monitored, and likely fluctuates less than external air temperatures due to the samplers being sheltered and the constant flow of water in the sewer lines. To evaluate any effect of air temperature during sample collection and shipment, ambient air temperatures in Edmonton (University of Alberta) and Calgary (University of Calgary) were monitored. Calgary data were collected and logged at the meteorological station located at the Calgary International Airport (World Meteorological Organization ID: 71877) operated by NAVCAN. Edmonton data were collected at the Blatchford weather station in Edmonton (World Meteorological Organization ID: 71157) operated by Environment and Climate Change Canada (Environment and Climate Change Canada [ECCC], 2022) as well as at the Meteorological Service of Canada. Weather data from 2020, 2021 and 2022 were accessed from the ECCC website enabling average daily temperature determinations to be used for correlation analyses.

To analyze the effect of ambient temperature during sampling and sample transport, the ambient outdoor air temperature averaged between Calgary and Edmonton and the difference in N1 levels estimated by each workflow was used. Mean average temperature of the two sites was analyzed against differences in N1 levels between the two workflows.

### 2.8 Data visualization and statistical analyses

**FIGURE 2:**
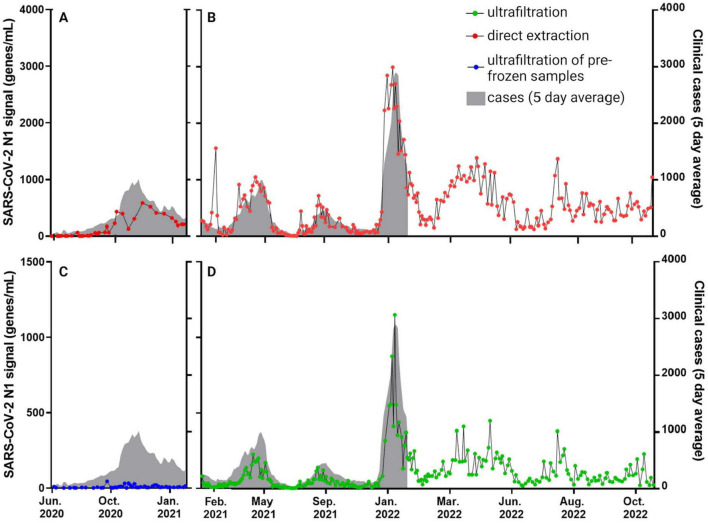
SARS-CoV-2 signals derived from assays for the N1 gene target abundance in raw influent wastewater collected from Calgary’s largest WWTP (i.e., WWTP-1; representing > 1 million people and 75% of the population of Calgary and surrounding communities). The same samples were split for processing by affinity column **(A,B)** and ultrafiltration **(C,D)**. Ultrafiltration samples were frozen prior to processing in the initial months of testing (June 2020 to January 2021 shown as blue circles in **(C)**; see also Supplementary Figure S6), then were processed without freezing as of February 2021 (green circles; **D**). Five-day rolling average clinically diagnosed COVID-19 cases are represented by the shaded gray areas (correlations between clinical and wastewater data are compiled in Table 2). Supplementary Figures S2–S5 show similar N1 datasets from the other WWTPs 2–5 in Calgary, Lethbridge and Fort McMurray, for different sub-sets of the 29-month time series shown here.

The R package “stats (v4.1.2)” was used for Spearman correlation analysis in R (v4.1.2). All graphics were generated either using GraphPad Prism (v 9.4.1), BioRender or basic embedded functions in R (v4.1.2). Due to a high degree of similarity between the N1 and N2 data, only N1 data is shown in Figure 2. For Spearman analysis, only paired data points (i.e., between two workflows, as shown in Figure 2) were considered, and all correlations were performed using log_10_-transformed data. Comparison between N1 data and clinically diagnosed COVID-19 cases used 5-day rolling averages of clinical cases from the day of comparison and the two prior and subsequent days. For analyzing ambient temperature effects, only data points where N1 gene abundance for the affinity column workflow was greater than for ultrafiltration workflow were considered (i.e., 336 out of 347 data points) when performing Spearman correlation.

**TABLE 1:** Spearman correlations for different workflow comparisons.

Gene target	Sample	Unfrozen ultrafiltration vs. unfrozen affinity column		Pre-frozen ultrafiltration vs. unfrozen affinity column	
		*r*	*p*	*r*	*P*
N1	WWTP-1	0.85	<0.0001	−0.045	0.90
	WWTP-2	0.69	<0.0001	0.52	0.20
	WWTP-3	0.71	<0.0001	0.39	0.21
	WWTP-4	0.67	<0.0001	NA	NA
	WWTP-5	0.70	<0.0001	NA	NA
N2	WWTP-1	0.87	<0.0001	0.65	0.034
	WWTP-2	0.67	<0.0001	−0.25	0.59
	WWTP-3	0.67	<0.0001	−0.51	0.094
	WWTP-4	0.63	<0.0001	NA	NA
	WWTP-5	0.71	<0.0001	NA	NA

**TABLE 2:** Spearman correlation between wastewater results and 5-day average clinically diagnosed COVID-19 cases.

Sample	Gene	Sample treatment	Method	Correlation with clinical cases	
				*r*	*p*
WWTP-1	N1	Unfrozen	Affinity column	0.82	<0.0001
		Pre-frozen	Ultrafiltration	0.49	0.0006
		Unfrozen	Ultrafiltration	0.86	<0.0001
	N2	Unfrozen	Affinity column	0.80	<0.0001
		Pre-frozen	Ultrafiltration	0.53	0.0002
		Unfrozen	Ultrafiltration	0.89	<0.0001
WWTP-2	N1	Unfrozen	Affinity column	0.85	<0.0001
		Pre-frozen	Ultrafiltration	0.62	0.1
		Unfrozen	Ultrafiltration	0.80	<0.0001
	N2	Unfrozen	Affinity column	0.77	<0.0001
		Pre-frozen	Ultrafiltration	0.32	0.4
		Unfrozen	Ultrafiltration	0.82	<0.0001
WWTP-3	N1	Unfrozen	Affinity column	0.81	<0.0001
		Pre-frozen	Ultrafiltration	0.55	0.06
		Unfrozen	Ultrafiltration	0.71	<0.0001
	N2	Unfrozen	Affinity column	0.74	<0.0001
		Pre-frozen	Ultrafiltration	−0.46	0.9
		Unfrozen	Ultrafiltration	0.76	<0.0001

## 3 Results

### 3.1 Longitudinal observations of wastewater SARS-CoV-2 N1 and N2 signals

Levels of SARS-CoV-2 N1 and N2 in wastewater samples detected using both workflows were similar to clinical patterns of COVID-19 prevalence in Calgary, Lethbridge and Fort McMurray during different waves of COVID-19 (Figure 2; Supplementary Figures S2–S5). Figure 2 highlights results based on SARS-CoV-2 N1 gene quantification from Calgary’s WWTP 1 showing both affinity column and ultrafiltration workflows. Results for N2 were strongly correlated with N1, as demonstrated previously (Acosta et al., 2021, 2022) therefore only N1 results are being reported. Alberta’s third, fourth and fifth waves of COVID-19 were evident in Calgary wastewater samples from April 2021, September 2021 and January 2022, respectively (Figures 2B,D). The latter was driven by the emergence of the Omicron variant in Alberta (Hubert et al., 2022) and representing the highest N1 levels in the entire dataset, i.e., roughly three times greater than in any of the prior waves. Clinical testing (gray shading; Figure 2) was scaled back to certain populations in late December 2021 during the fifth wave (e.g., to individuals whose COVID-19 disease would qualify for antiviral therapy, those with severe illness presenting to emergency departments, and healthcare workers) (Alberta, 2023). In this context of limited clinical testing, wastewater data provided a reliable estimation of total disease burden than clinical data during subsequent waves of Omicron infections throughout 2022 (Figure 2).

### 3.2 Freezing wastewater prior to analysis

**FIGURE 3:**
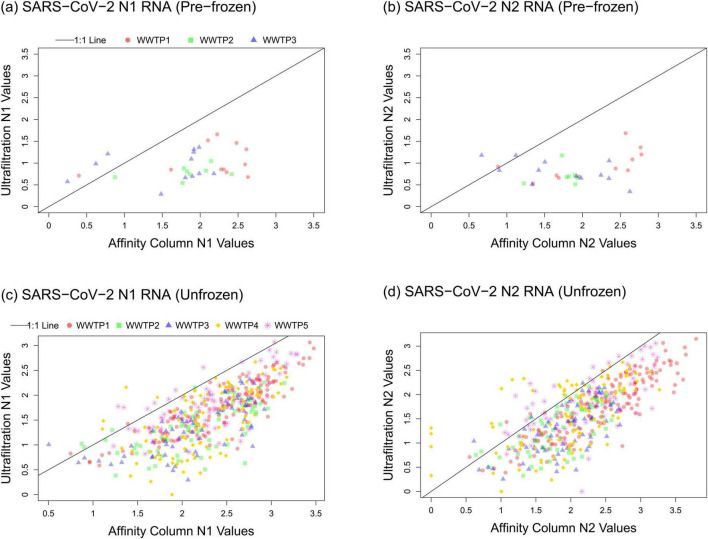
Relationships between the SARS-CoV-2 data obtained using affinity column and ultrafiltration workflows for **(A)** N1 pre-frozen, **(B)** N2 pre-frozen, **(C)** N1 unfrozen, and **(D)** N2 unfrozen samples. Log_10_-transformed gene abundance values are plotted for the ultrafiltration workflow (y-axes) and the affinity column workflow (x-axes).

Correlation between workflows was low or insignificant for the period when ultrafiltration samples were frozen prior to being thawed for processing (June 2020 to January 2021; Table 1). Despite significant correlation between clinical cases in Calgary and ultrafiltration data using either frozen (*p* = 0.0006) or unfrozen (*p* < 0.0001) samples from WWTP 1 during this time frame, comparing results between affinity column and ultrafiltration workflows revealed much stronger agreement when all samples were unfrozen after January 2021 (*r* ≥ 0.80 compared to *r* ≤ 0.65 when ultrafiltration samples were pre-frozen). This is likely due to deterioration of SARS-CoV-2 signal during freeze-thaw processes (variable N1 signals during this period are shown in Supplementary Figure S6). Accordingly, differences in N1 gene abundances between the two workflows were larger for pre-frozen ultrafiltration samples compared to unfrozen ultrafiltration samples (Figure 2). Based on these observations, workflow comparisons focused on the period after January 2021 (Figure 3; Supplementary Figures S2–S5).

### 3.3 Comparing affinity column and ultrafiltration results

Spearman correlation analysis was performed for N1 results measured by each workflow on samples collected after January 2021 (Table 1). The strongest correlation between workflows was observed for WWTP 1 (*r* = 0.85) which served the largest population (roughly 1.1 million people). Good correlation was also observed for WWTP 2 (*r* = 0.69) and WWTP 3 (*r* = 0.71) that both serve a smaller catchment within Calgary corresponding to roughly 363,000 people. Good correlations were also observed for WWTP 4 in Lethbridge (*r* = 0.67) and WWTP 5 in Fort McMurray (r = 0.70) which collect all of the wastewater from those municipalities (76,006 and 101,799 people, respectively).

The performance of either workflow as a predictor of clinically diagnosed COVID-19 cases was very similar. N1 and N2 signals produced by both affinity column and ultrafiltration workflows correlated with clinical cases for WWTP 1 (*r* ≥ 0.80 and *p* < 0.0001; Table 2) when reliable clinical data was available up until the end of 2021. The ultrafiltration workflow using RNA standards generally produced lower gene abundance values than the affinity column workflow using DNA standards for all monitoring sites (Figure 3), as has been reported before (Hou et al., 2010; Chik et al., 2021), but this does not impact correlations of either dataset with clinical cases (Table 2) for unfrozen samples.

### 3.4 Assessing effects of ambient temperature on SARS-CoV-2 quantification

**FIGURE 4:**
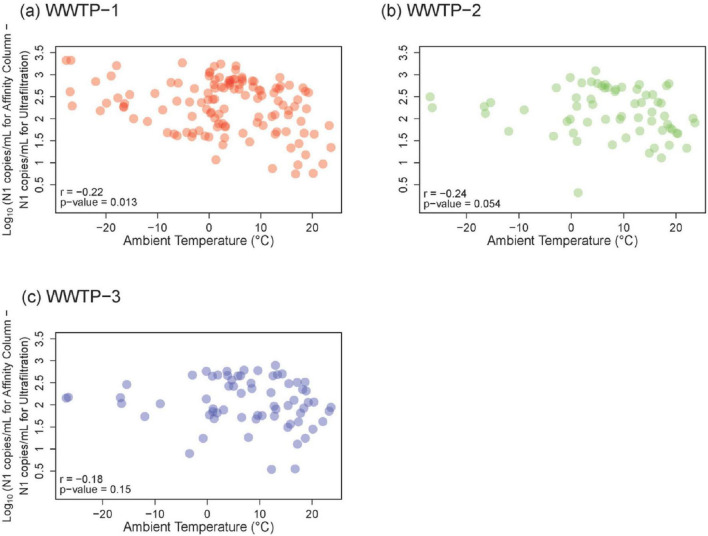
Relationship between ambient temperature and Log_10_-transformed N1 gene abundance data between two workflows for **(A)** WWTP-1, **(B)** WWTP-2, and **(C)** WWTP-3 samples. Ultrafiltration values were subtracted from affinity column values and the difference is plotted following Log_10_-transformation. Unfrozen samples from both workflows were used for these analyses, which correspond to January 2021 to November 2022 for WWTP-1, and January 2021 to September 2021 for WWTP-2 and WWTP-3. Spearman correlation *r-* and *p*-values are shown in the lower left of each panel.

Longitudinal comparisons allow external factors like seasonal temperature to be assessed and to understand correlations at different stages of the pandemic. Samples from Calgary WWTPs 1–3 were exposed to longer storage and transit associated with the ultrafiltration workflow performed at the University of Alberta (in Edmonton, 300 km away from the sampling locations) than affinity column processing performed at the University of Calgary (in the same city). N1 gene abundance data were considered together with ambient air temperature to examine whether sample storage temperature during the extra transport time (overnight shipping from Calgary to Edmonton, compared to < 8 h transit for samples analyzed in Calgary) impacts results. Spearman correlation analysis between ambient temperature and the difference in gene abundance between the workflows revealed slightly more discordance between ultrafiltration and affinity column results associated with lower ambient temperatures (Figure 4). The highest Spearman correlation between gene abundance difference and colder temperature was observed for WWTP 1 (*r* = −0.22, *p* = 0.013), followed by WWTP 2 (*r* = −0.24, *p* = 0.054), and WWTP 3 (*r* = −0.18, *p* = 0.15). These results do not point to a very strong transport temperature effect overall. This comparison was not performed for WWTP 4 and WWTP 5, since samples from both sites had to be shipped to different cities for both ultrafiltration and affinity column processing.

### 3.5 PMMoV normalization effects

Values for pepper mild mottle virus (PMMoV) in wastewater samples were generally an order of magnitude higher for samples processed by ultrafiltration compared to using affinity columns. Accordingly, correlations between affinity column wastewater (WWTP 1; N1 gene) and clinical cases were markedly lower with PMMoV normalization (*r* = 0.82 dropping to *r* = 0.56), whereas little difference was observed when using ultrafiltration results with and without PMMoV normalization (*r* = 0.81 and 0.86, respectively) (Supplementary Table S14). PMMoV levels fluctuated over time for samples from all of the WWTPs. Generally, very low PMMoV levels were measured from affinity column processed samples, though periodic increases in this signal were noted. Similar observations were made across all WWTPs (Supplementary Figures S7–S11).

## 4 Discussion

The COVID-19 pandemic resulted in large-scale innovation of wastewater-based surveillance strategies in municipalities around the world. These efforts began in 2020 as the unfolding pandemic challenged healthcare systems. In this context, WBS programs understandably did not emerge in a coordinated way. Accordingly, despite the success of WBS around the world, there is no universally standard approach for making these measurements. The example of Alberta, Canada demonstrates that two independent teams based in two separate large urban centers adopted different methods for measuring and reporting SARS-CoV-2 trends in municipal wastewater samples. In this case, both testing labs had access to samples from the same WWTPs, enabling this comparative study. This revealed that two different workflows applied consistently—despite giving rise to different viral RNA quantification values—both reliably revealed comparable trends in local COVID-19 disease burden during the pandemic. These workflows used fundamentally different methods but proved to be equally valuable and useful for stakeholders.

While the extraction efficiency of SARS-CoV-2 may differ between affinity column and ultrafiltration methods, and qPCR efficiencies will differ when using DNA or RNA standards, the two workflows did not give rise to a significant difference in overall trends (Figure 2; Table 2). Several waves of COVID-19 were reliably identified and forecast by both workflows during this 29-month longitudinal study. Many other studies have successfully performed longitudinal monitoring of diseases occurrences while employing various laboratory protocols. Several studies have compared diverse workflows. This has included examining grab samples vs. using composite samplers (Medema et al., 2020; Randazzo et al., 2020) or comparing different sample concentration strategies and RNA extraction methods, e.g., ultrafiltration, affinity columns or Al(OH)_3_ adsorption-precipitation (Hata et al., 2021; Nemudryi et al., 2020; Randazzo et al., 2020). Reliance on different molecular quantitative analysis tools has also been compared, e.g., RT digital PCR or RT-qPCR (Ahmed et al., 2022; Vadde et al., 2022). The consensus from these studies is that significant correlation between clinical cases and SARS-CoV-2 RNA levels in wastewater can be achieved by different workflows. This does not mean gene abundance values will not differ when using different approaches, as was observed in this study (see Figures 2C,D). It is therefore important not to rely on or compare exact quantified values, but rather to focus on resulting trends as reliable predictors of the trajectory of viral burden in the community being tested. In the case of the present study, both workflows performed similarly well in anticipating clinical cases using unfrozen samples (*r* ≥ 0.71 and *p* < 0.0001; Table 2).

This is not to say that improvements to the workflows presented here and elsewhere should not be pursued. For example, reliance on commercially available DNA standards (Workflow A in this study) is susceptible to variability between manufacturer batches as well as incorrectly estimated concentrations if batches are not certified (Bivins et al., 2021). Indeed, we observed batch to batch variation DNA plasmid standard concentrations (up to fivefold) as verified using digital PCR (unpublished data). This highlights that absolute quantification by digital PCR is an attractive alternative to RT-qPCR for future WBS applications, as digital PCR does not rely on standard curves (Mirabile et al., 2024; Tiwari et al., 2022) that can pose challenges (Schmidt et al., 2023).

Streamlining of various aspects of WBS workflows into a common or standardized approach is intuitive and perhaps inevitable. Nevertheless, these efforts are unlikely to overcome the inherent physicochemical differences in the wastewater matrix from different WWTPs, which should ultimately prevent samples from different sewersheds from being directly compared regardless of the workflow(s) employed. These differences include proportions of urban and industrial inputs, the influence of stormwater dilution in some but not all instances, and variations in the amounts of different PCR inhibitors present in the samples (Muttamara, 1996). While it may be tempting to pursue standardization to allow better comparisons between laboratories and different sampling locations, this objective should be approached with caution. On the other hand, the results of the present study show that even when different methods are applied in a consistent manner, remarkably comparable trends are generated.

Extended longitudinal analysis allowed external ambient temperature to be examined as a potentially confounding factor that impacts the quality of target RNA in the samples and its quantification. This was especially evident with intentional freezing of raw wastewater samples prior to RNA isolation using ultrafiltration (Workflow B), which resulted in much lower signals from RT-qPCR analysis (Figures 2A,B, 3). Further assessment of temperature during the extra storage time associated with the ultrafiltration workflow here gave more nuanced results. Divergence in SARS-CoV-2 quantification values between the two workflows were greatest for samples that encountered very cold ambient temperatures below -20°C (Figure 4). This could potentially lead to unintentional freezing of raw wastewater following sample exposure to ambient air (e.g., during loading or unloading, or depending on where wastewater autosamplers are situated for 24-h composite sample collection). Inadvertent freeze-thaw events during storage and prior to sample processing could produce similar effects on the RT-qPCR signal as the intentional freezing shown in Supplementary Figure S6. Maintaining samples unfrozen at low temperature should minimize changes to RNA levels and allow associated disease trends to be tracked with high fidelity. As has been highlighted in other studies (Huge et al., 2022; Markt et al., 2021; Simpson et al., 2021) it is recommended to process wastewater samples without freezing, and as quickly as reasonably possible after collection. If freezing is unavoidable due to various logistics (e.g., very extended time between collection and processing during periods with low ambient temperature), freezing effects can potentially be minimized by adding a preservative (salt, buffer, polyethylene glycol) or by removal of water via concentration (i.e., charged membrane filtration) prior to freezing (Steele et al., 2021). The results presented here underscore that any such interventions should be applied consistently to all samples, not just in instances where freezing temperatures are expected. Indeed, the goal is to maintain a consistent approach that enables longitudinal comparison of samples from the same sewershed. Similar considerations should be incorporated into the timing of sampling and associated transport. Since the timing may vary, e.g., for analysis in Calgary of samples obtained nearby (Calgary WWTPs 1–3) or farther away such as 250 km (Lethbridge WWTP 4) or 700 km (Fort McMurray WWTP 5), maintaining high fidelity trends depends on consistent sample treatment with respect to timing and protocol. This way the most important information being generated by this kind of testing—local disease trends within a sewershed’s corresponding population and community—can be monitored reliably.

WBS programs may prioritize certain advantages of specific steps or methodologies in determining an overall workflow. The affinity column method used here (Whitney et al., 2021) does not require additional RNA extraction but may not as rigorously capture and incorporate viruses with capsids that have highly resilient structures. This limitation will be important in ongoing and future applications of this technology that move beyond SARS-CoV-2. Ultrafiltration requires a large benchtop centrifuge, presenting a limitation to sample throughput, but by enabling viral capture based on size exclusion this method can be more inclusive of a wider variety of capsid structures. Overall, the affinity column method is about half the per-sample cost of the ultrafiltration method. Extraction efficiency of PMMoV, a well-known indicator of human origin fecal contamination (Kitajima et al., 2018), was lower using affinity columns than with ultrafiltration, resulting in values differing by an order of magnitude (Supplementary Figures S7–S11). As a consequence, significantly weakened correlation between clinical cases and N1 signals in wastewater after normalization by PMMoV from affinity columns relative to ultrafiltration results was observed, highlighting that PMMoV normalization is not advisable when using affinity columns (Supplementary Tables S14, S15). This limitation may be due to the relatively poor extraction efficiency of PMMoV virions by affinity column processing steps, which appear to be aggressive enough to lyse membranes of the SARS-CoV-2 envelope but less effective at disrupting viruses with more sturdy capsids like PMMoV (Jafferali et al., 2021; Kitajima et al., 2020; Symonds et al., 2018). Viral concentration and subsequent RNA extraction steps found in the ultrafiltration workflow likely contribute to more robust PMMoV lysis and recovery, which has proven to be valuable in some contexts (D’Aoust et al., 2021). Other factors in wastewater that may enable fecal normalization for the contributing human population to a given sewershed, such as human *Bacteroides* strain HF183 (Feng et al., 2021) may be preferable when using affinity columns. That being said, as has been consistently reported in the literature (Feng et al., 2021; Greenwald et al., 2021; Mitranescu et al., 2022), our results reiterate that raw wastewater provides the best correlations with clinical data and for wastewater data compared between different workflows (Figure 2; Table 2).

An advantage of the affinity column direct extraction method used here is that both solid and liquid associated viruses are incorporated owing to the lysis step occurring before the removal of solids (Logisz and Hovis, 2005). This direct RNA extraction enabled by affinity column processing also benefits from preventing the loss of free nucleic acids or solids-associated viral fragments that may not be retained during ultrafiltration (Whitney et al., 2021). These factors may contribute to the higher RT-qPCR values for affinity column results, although other variables including losses associated with the additional RNA extraction step associated with ultrafiltration, or the two different workflows using different RT-qPCR standards, also influence the difference observed here between direct extraction and ultrafiltration.

## 5 Conclusion

This study provides a 29-month experimental dataset assessing the longitudinal performance of two WBS workflows applied to samples from five WWTPs across three municipalities. Strong correlations highlight how either workflow is capable of effectively monitoring COVID-19 trends if applied consistently throughout the monitoring period. Despite methodological differences, both affinity column and ultrafiltration workflows were used successfully for monitoring three municipalities for over 2 years.

## Data Availability

The original contributions presented in the study are included in the article/Supplementary material, further inquiries can be directed to the corresponding authors.
