# Supplementary material for: Consistent trends from different methods for monitoring SARS-CoV-2 in urban wastewater during a 29-month longitudinal study
Source: Front Microbiol. 2025 Jun 20;16:1547831. doi: 10.3389/fmicb.2025.1547831 (PMC12226558; doi:10.3389/fmicb.2025.1547831)
Supplement: Supplementary file 1 [file Data_Sheet_1.pdf]

Consistent trends from different methods for monitoring SARS-CoV-2 in urban wastewater during a 29-month longitudinal study

Supplementary Material

S1. Geographic distribution of WWTPs.

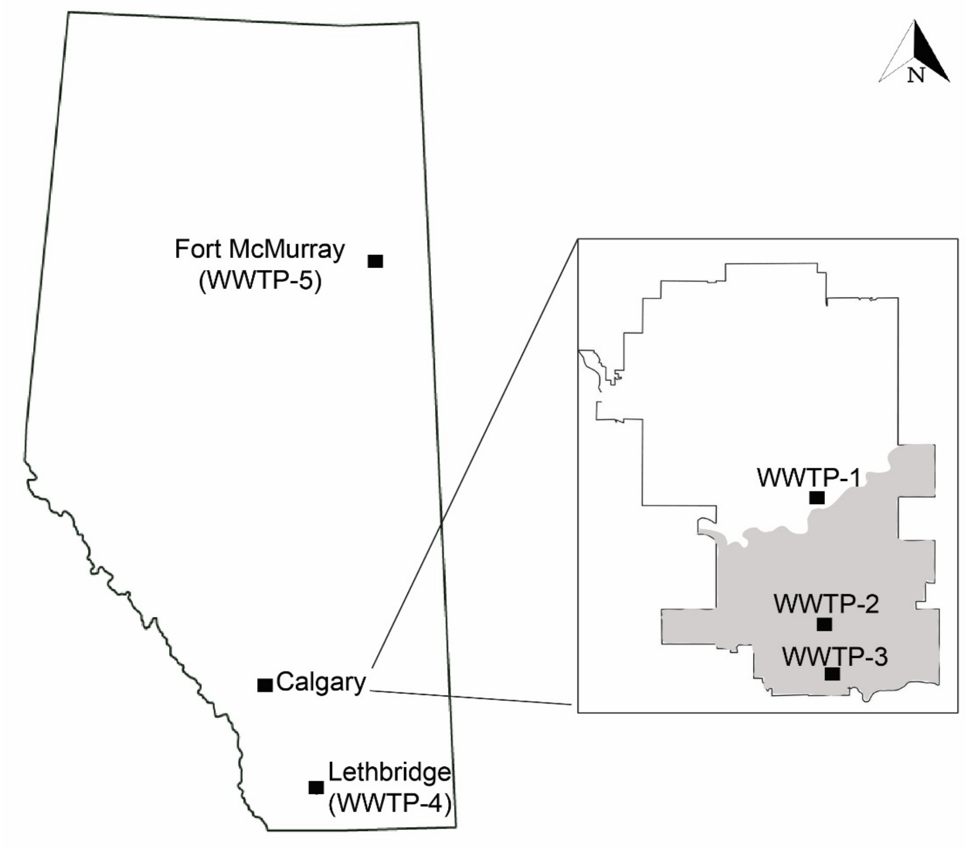

Figure S1: Location of the five wastewater treatment plants sampled in this study. The province of Alberta is shown on the left, with the inset showing the city of Calgary.

S2. RT-qPCR assay specifications

Table S1: RT-qPCR primers and probes used for detection assays

Target	Forward primer	Reverse Primer	Probe
N1	GAC CCC AAA ATC AGC GAA AT	TCT GGT TAC TGC CAG TTG AAT CTG	ACC CCG CAT TAC GTT TGG TGG ACC
N2	TTA CAA ACA TTG GCC GCA AA	GCG CGA CAT TCC GAA GAA	ACA ATT TGC CCC CAG CGC TTC AG
BCoV	CTG GAA GTT GGT GGA GTT	ATT ATC GGC CTA ACA TAC ATC	CCT TCA TAT CTA TAC ACA TCA AGT TGT T
PMMoV	GAG TGG TTT GAC CTT AAC GTT TGA	TTG TCG GTT GCA ATG CAA GT	CCT ACC GAA GCA AAT G
HCoV229E	TTC CGA CGT GCT CGA ACT TT	CCA ACA CGG TTG TGA CAG TGA	TCC TGA GGT CAA TGC A

S2.1. Affinity column RT-qPCR assay information

Table S2: SARS-CoV-2 N1 and N2 assays.

Constituent	Volume (µl)
Sample	5
TaqMan™ Fast Virus 1-Step Master Mix	5
Forward and reverse primers (10 000 nM)	1
Probe (2500 nM)	1
Nuclease-free water	8

Table S3: SARS-CoV-2 N1 and N2 assay PCR conditions.

Stage	Cycles	Time	Temperature (℃)
1	1	00:05:00	50
2	1	00:0020	95
3	45	00:00:03	95
3	45	00:00:30	55

Table S4: BCoV and PMMoV multiplexed assay.

Constituent	Volume (µl)
Sample	4
TaqPath™ Fast Virus 1-Step Master Mix	5
Forward and reverse BCoV primers (4000 nM)	1
Forward and reverse PMMoV primers (8000 nM)	1
BCoV probe (2500 nM)	1
PMMoV probe (4000nM)	1
Nuclease-free water	7

Table S5: BCoV and PMMoV multiplexed assay PCR conditions.

Stage	Cycles	Time	Temperature (℃)
1	1	00:02:00	25
2	1	00:10:00	53
3	1	00:02:00	95
4	40	00:00:03	95
4	40	00:00:30	60

S2.2. Ultrafiltration RT-qPCR assay information

Table S6: SARS-CoV-2 N1 and N2 assays. Final volume of 10 µl.

Constituent	Volume (µl)
TaqMan fast virus one-step MM (4X)	2.5
Forward primers (20 µM)	0.4
Reverse primers (20 µM)	0.4
Probe (10 µM)	0.2
Nuclease-free water	1.5
RNA	5

Table S7: SARS-CoV-2 N1 and N2 assay cycling conditions.

Stage	Cycles	Time	Temperature (℃)
1	1	00:05:00	50
2	1	00:00:20	95
3	45	00:00:03	95
3	45	00:00:30	60

Table S8: PMMoV assay.

Constituent	Volume (µl)
TaqMan fast virus one-step MM (4X)	2.5
Forward primers (10 µM)	0.5
Reverse primers (10 µM)	0.5
Probe (10 µM)	0.2
Nuclease-free water	1.3
RNA	5

Table S9: PMMoV assay cycling conditions.

Stage	Cycles	Time	Temperature (℃)
1	1	00:05:00	50
2	1	00:00:20	95
3	45	00:00:03	95
3	45	00:00:30	60

Table S10: HCoV229E assay.

Constituent	Volume (µl)
TaqMan fast virus one-step MM (4X)	2.5
Forward primers (10 µM)	0.4
Reverse primers (10 µM)	0.4
Probe (10 µM)	0.2
Nuclease-free water	1.5
RNA	5

Table S11: HCoV229E assay cycling conditions.

Stage	Cycles	Time	Temperature (℃)
1	1	00:05:00	50
2	1	00:00:20	95
3	45	00:00:03	95
3	45	00:00:30	60

Table S12: Salmon DNA assay.

Constituent	Volume (µl)
TaqMan fast universal PCR MM (2X)	5
Primers and probe mix (18 µM F/R & 5 µM P)	0.5
Nuclease-free water	2
RNA	2.5

Table S13: Salmon DNA assay cycling conditions.

Stage	Cycles	Time	Temperature (℃)
1	1	00:00:20	95
3	45	00:00:03	95
3	45	00:00:30	60

S3. SARS-CoV-2 N1 signal from WWTPs 2-5

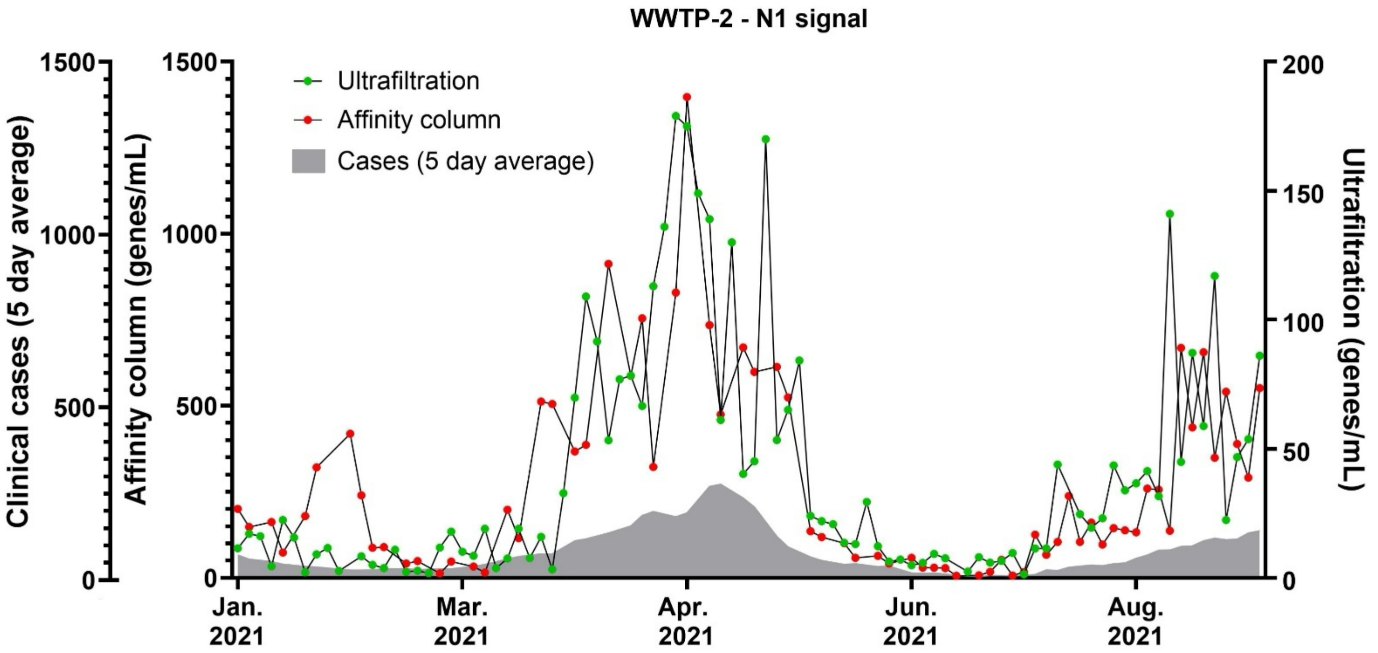

Figure S2: SARS-CoV-2 N1 signals from raw influent wastewater sampled at WWTP-2 (i.e., sampling >360,000 people) in Calgary. Data was collected between January 2021 and September 2021. The same samples were split for processing by affinity column (red circles) and ultrafiltration (green circles). Five-day rolling average clinically diagnosed COVID-19 cases are represented by the shaded grey areas (correlations between clinical and wastewater data are compiled in Table 2).

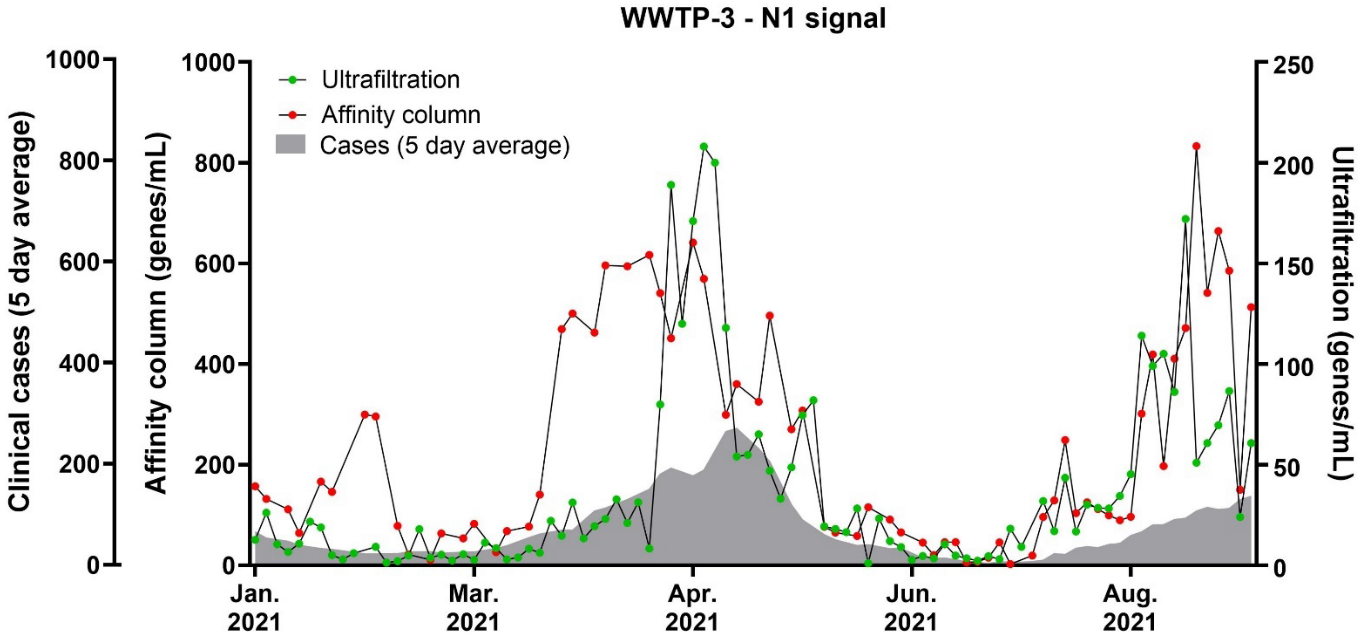

Figure S3: SARS-CoV-2 N1 signals in raw influent wastewater sampled at WWTP-3 (i.e., sampling >360,000 people) in Calgary. Data was collected between January 2021 and September 2021. The same samples were split for processing by affinity column (red circles) and ultrafiltration (green circles). Five-day rolling average clinically diagnosed COVID-19 cases are represented by the shaded grey areas (correlations between clinical and wastewater data are compiled in Table 2).

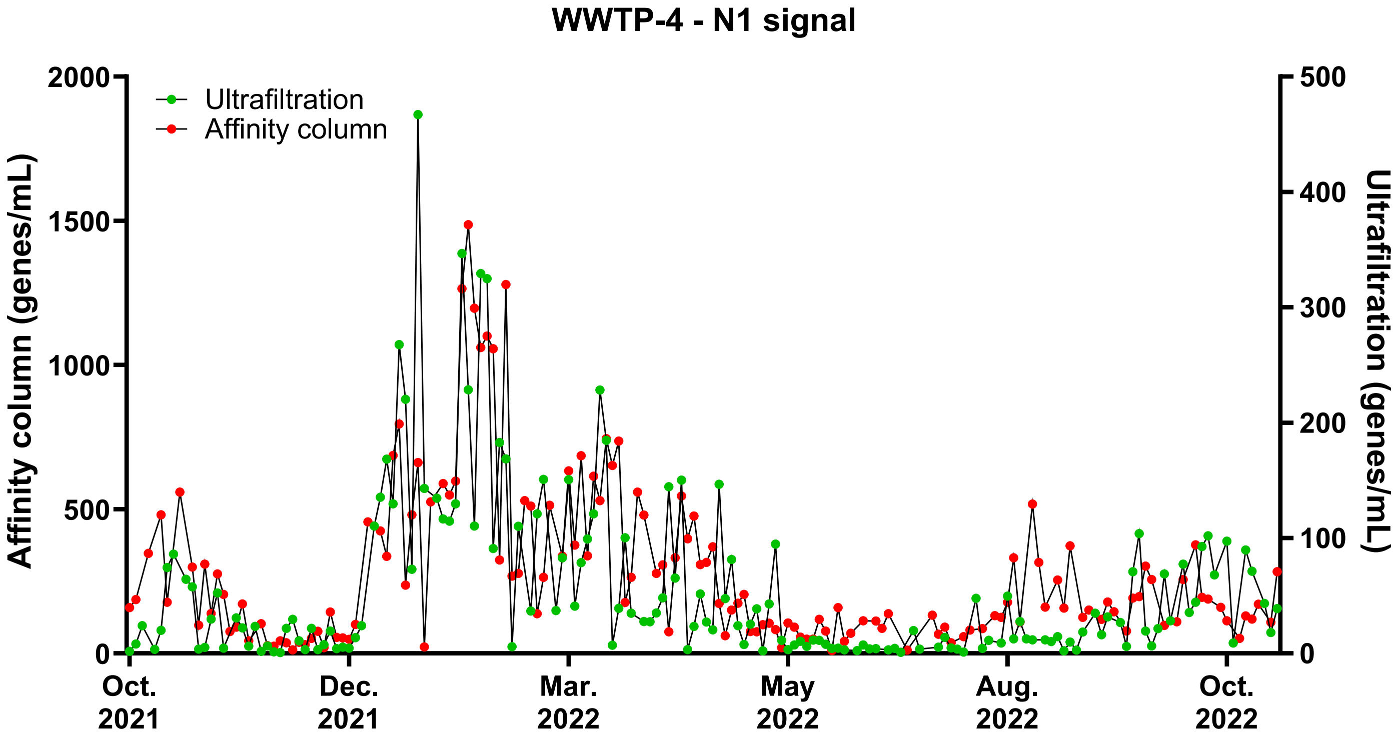

Figure S4: SARS-CoV-2 N1 signals in raw influent wastewater sampled at WWTP-4 (i.e., sampling >100,000 people) in Lethbridge. Data was collected between October 2021 and November 2022. The same samples were split for processing by affinity column (red circles) and ultrafiltration (green circles).

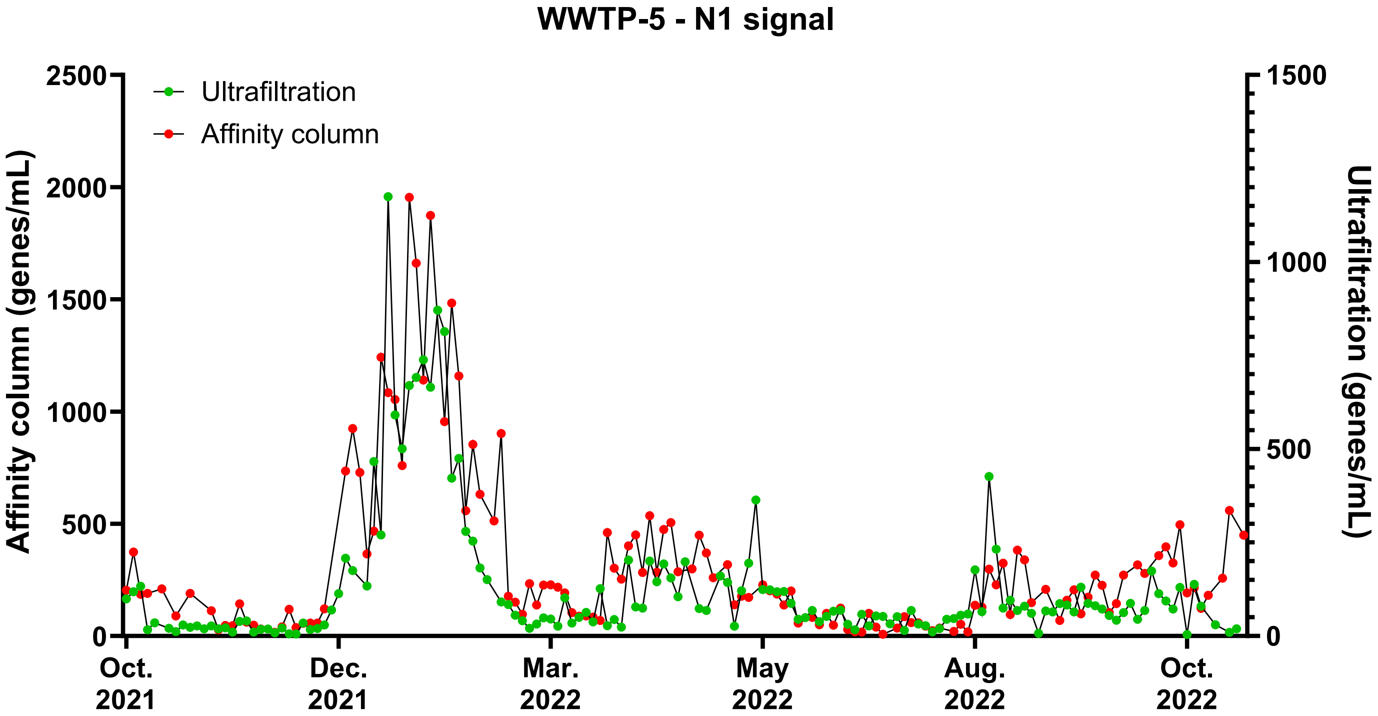

Figure S5: SARS-CoV-2 N1 signals in raw influent wastewater sampled at WWTP-5 (i.e., sampling >76,000 people) in Fort McMurray. Data was collected between October 2021 and November 2022. The same samples were split for processing by affinity column (red circles) and ultrafiltration (green circles).

S4. Frozen samples processed by ultrafiltration

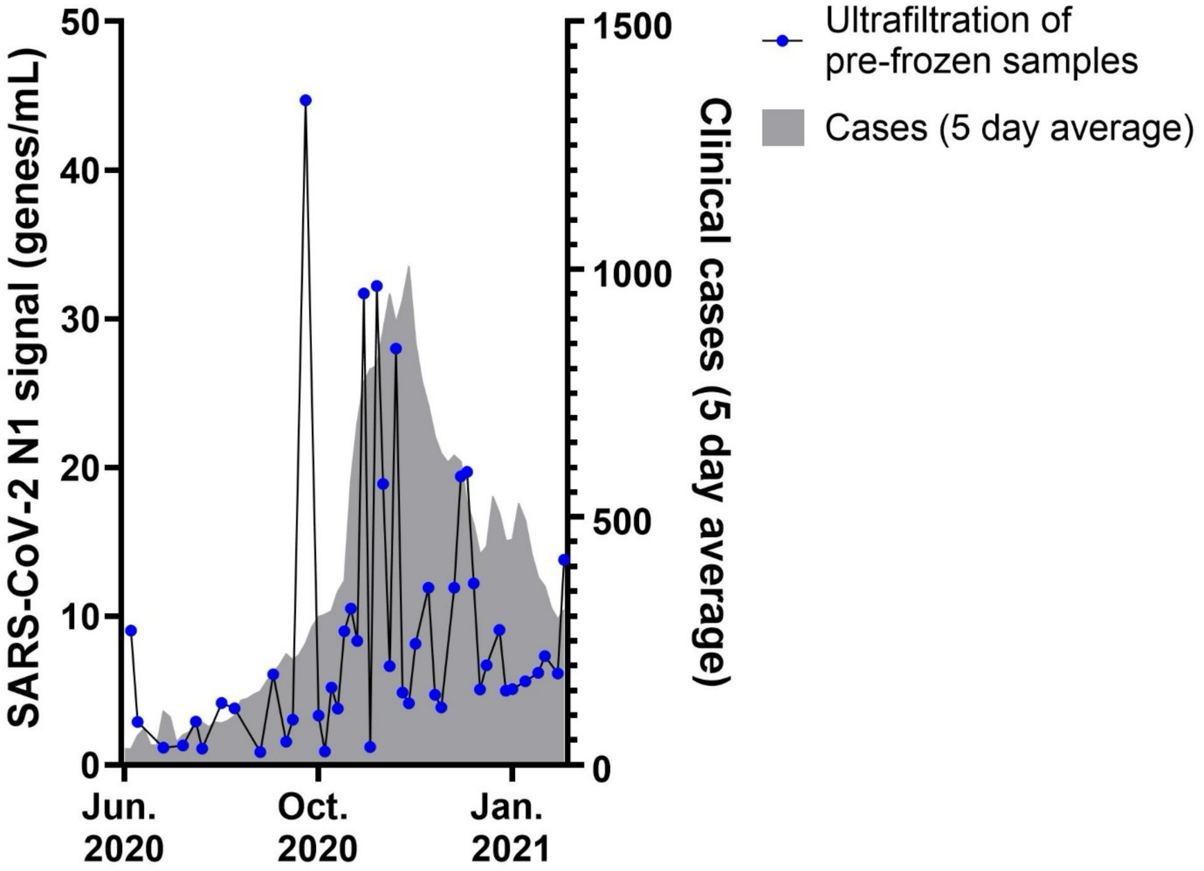

Figure S6: SARS-CoV-2 N1 signal from raw influent wastewater sampled at WWTP-1 (i.e., sampling >1 million people) in Calgary. Data was collected between June 2020 and January 2021. Samples were frozen prior to being processed by ultrafiltration (blue circles). Five-day rolling average clinically diagnosed COVID-19 cases are represented by the shaded grey area.

S5. PMMoV signal

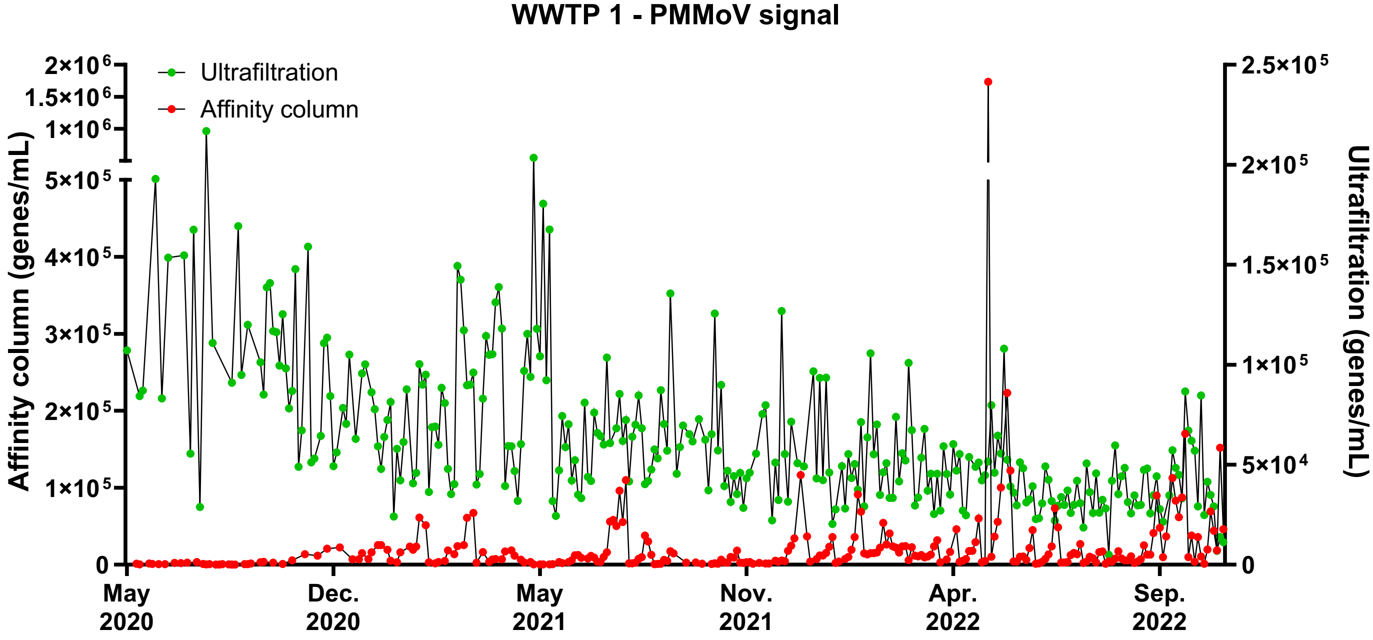

Figure S7: PMMoV signals in raw influent wastewater sampled at WWTP-1 (i.e., sampling >1 million people) in Calgary. Data was collected between May 2020 and November 2022. The same samples were split for processing by affinity column (red circles) and ultrafiltration (green circles).

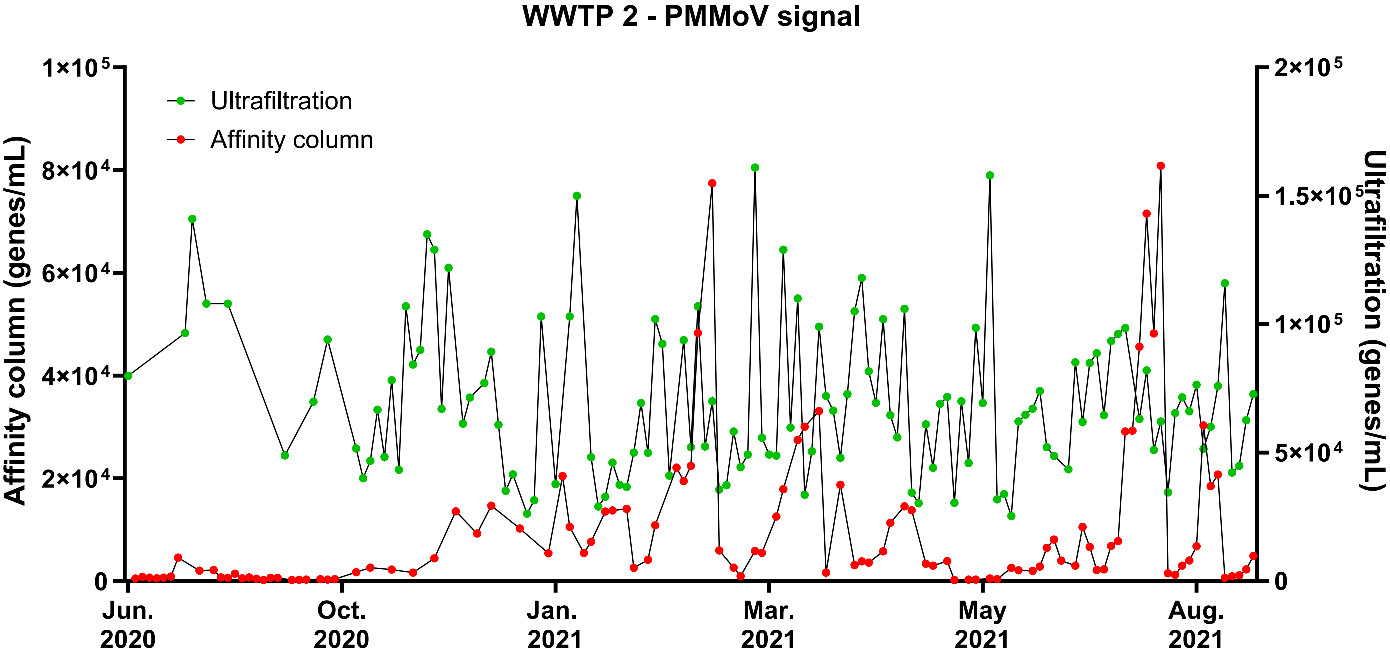

Figure S8: PMMoV signals in raw influent wastewater sampled at WWTP-2 (i.e., sampling >360,000 people) in Calgary. Data was collected between June 2020 and September 2021. The same samples were split for processing by affinity column (red circles) and ultrafiltration (green circles).

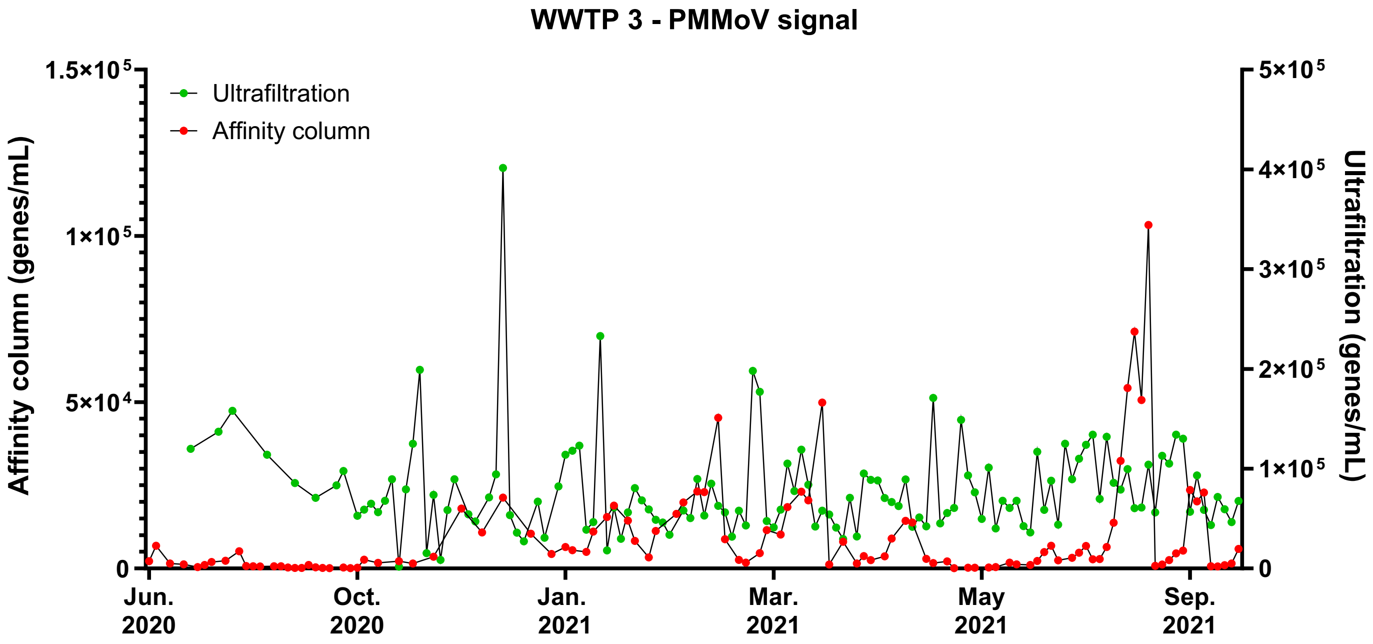

Figure S9: PMMoV signals in raw influent wastewater sampled at WWTP-3 (i.e., sampling >360,000 people) in Calgary. Data was collected between June 2020 and September 2021. The same samples were split for processing by affinity column (red circles) and ultrafiltration (green circles).

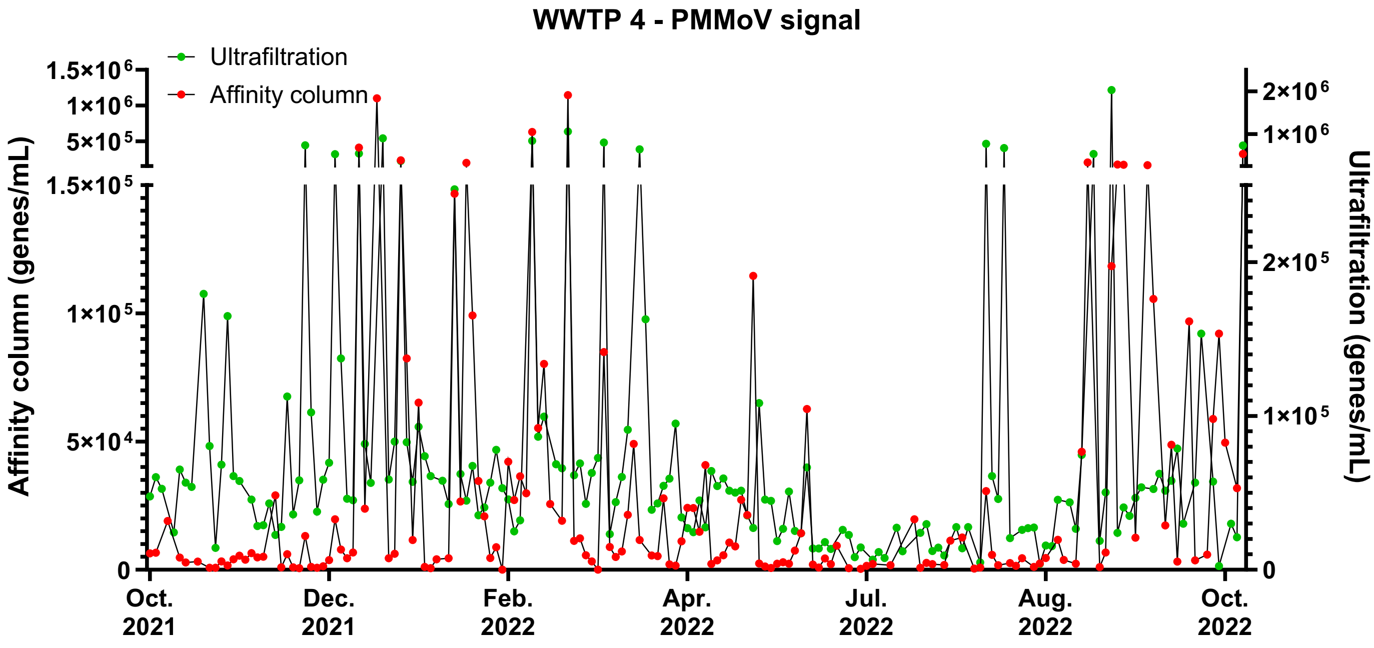

Figure S10: PMMoV signals in raw influent wastewater sampled at WWTP-4 (i.e., sampling >100,000 people) in Lethbridge. Data was collected between October 2021 and November 2022. The same samples were split for processing by affinity column (red circles) and ultrafiltration (green circles).

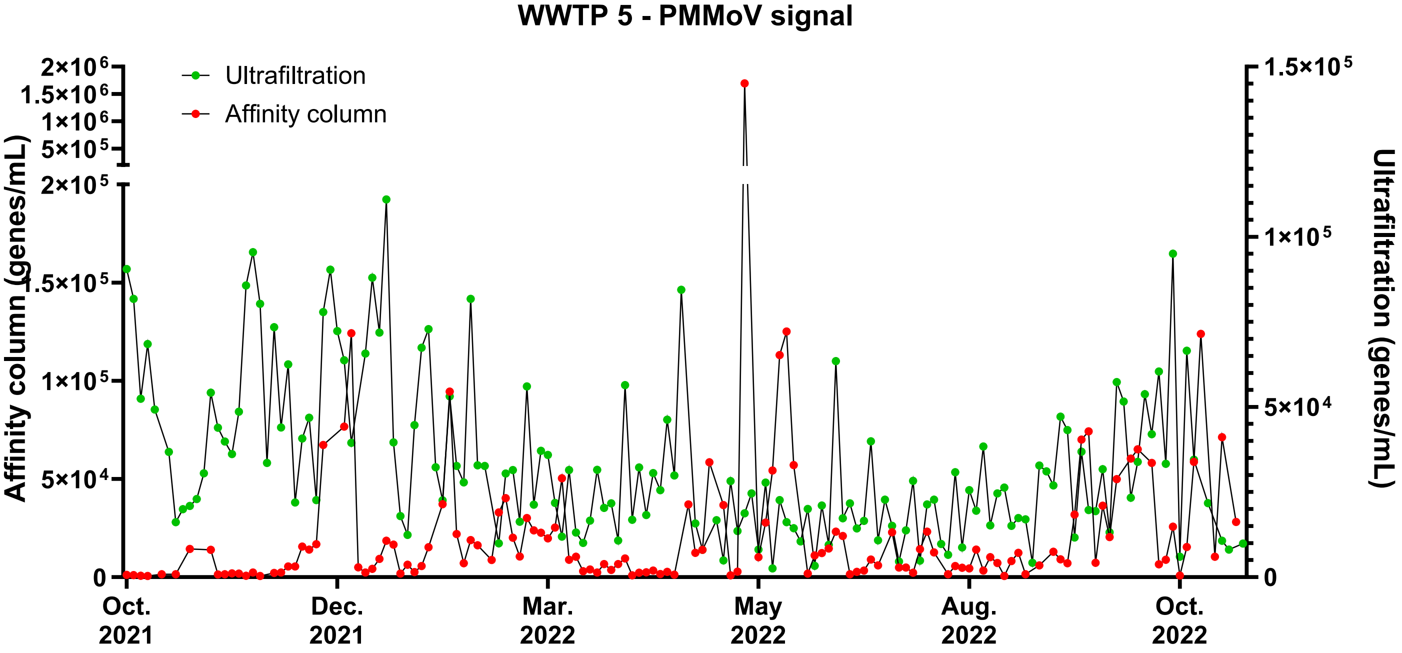

Figure S11: PMMoV signals in raw influent wastewater sampled at WWTP-5 (i.e., sampling >76,000 people) in Fort McMurray. Data was collected between October 2021 and November 2022. The same samples were split for processing by affinity column (red circles) and ultrafiltration (green circles).

S6. PMMoV normalization correlation analyses

Table S14: Spearman correlation of affinity column (AC) or ultrafiltration (UF) N1 results with clinical cases, with and without PMMoV normalization.

Site	Method	Clinical case correlation with PMMoV normalization		Clinical case correlation without PMMoV normalization	
		**r**	**p**	**r**	**p**
WWTP-1	AC	0.56	<0.0001	0.82	<0.0001
	UF	0.81	<0.0001	0.86	<0.0001
WWTP-2	AC	0.70	<0.0001	0.85	<0.0001
	UF	0.81	<0.0001	0.80	<0.0001
WWTP-3	AC	0.65	<0.0001	0.81	<0.0001
	UF	0.78	<0.0001	0.71	<0.0001

Table S15: Spearman correlation between affinity column and ultrafiltration N1 results with and without PMMoV normalization.

Site	Method comparison with PMMoV normalization		Method comparison without PMMoV normalization	
	**r**	**p**	**r**	**p**
WWTP-1	0.36	<0.0001	0.85	<0.0001
WWTP-2	0.58	<0.0001	0.69	<0.0001
WWTP-3	0.57	<0.0001	0.71	<0.0001
WWTP-4	0.23	0.01	0.67	<0.0001
WWTP-5	0.14	0.12	0.70	<0.0001
